# Finite Element Analysis of Lightning Damage Factors Based on Carbon Fiber Reinforced Polymer

**DOI:** 10.3390/ma14185210

**Published:** 2021-09-10

**Authors:** Yansong Zhu, Yueke Ming, Ben Wang, Yugang Duan, Hong Xiao, Chenping Zhang, Jinru Sun, Xiangyu Tian

**Affiliations:** 1 State Key Laboratory for Manufacturing Systems Engineering, Xi’an Jiaotong University, Xi’an 710049, China; xjtu_zys@stu.xjtu.edu.cn (Y.Z.); mingyueke@foxmail.com (Y.M.); ygduan@mail.xjtu.edu.cn (Y.D.); xiaohongjxr@xjtu.edu.cn (H.X.); zcp0824@stu.xjtu.edu.cn (C.Z.); 2 State Key Laboratory of Electrical Insulation and Power Equipment, Xi’an Jiaotong University, Xi’an 710049, China; txiangyu@stu.xjtu.edu.cn

**Keywords:** carbon-fiber-reinforced polymer, lightning strike, finite element, influencing factors, damage degree

## Abstract

While carbon-fiber-reinforced polymers (CFRPs) are widely used in the aerospace industry, they are not able to disperse current from lightning strikes because their conductivity is relatively low compared to metallic materials. As such, the undispersed current can cause the vaporization or delamination of the composites, threatening aircraft safety. In this paper, finite element models of lightning damage to CFRPs were established using commercial finite element analysis software, Abaqus, with the user-defined subroutines USDFLD and HEAVEL. The influences of factors such as the structural geometry, laminate sequence, and intrinsic properties of CFRPs on the degree of damage to the composites are further discussed. The results showed that when a current from lightning is applied to the CFRP surface, it mainly disperses along the fiber direction in the outermost layer. As the length of the CFRP increases, the injected current has a longer residence time in the material due to the increased current exporting distance. Consequently, larger amounts of current accumulate on the surface, eventually leading to more severe damage to the CFRP. This damage can be alleviated by increasing the thickness of the CFRP, as the greater overall resistance makes the CFRP a better insulator against the imposed current. This study also found that the damaged area increased as the angle between the first two layers increased, whereas the depth of the damage decreased due to the current dispersion between the first two layers. The analysis of the electrical conductivity of the composite suggested that damage in the fiber direction will be markedly reduced if the conductivity in the vertical fiber direction increases approximately up to the conductivity of the fiber direction. Moreover, increasing the thermal conductivity along the fiber direction will accelerate the heat dissipation process after the lightning strike, but the influence of the improved thermal conductivity on the extent of the lightning damage is less significant than that of the electrical conductivity.

## 1. Introduction

In the aircraft industry, carbon-fiber-reinforced polymers (CFRPs) have gradually replaced traditional metallic materials in parts such as skins and fairings due to their excellent mechanical properties, including their high specific strength and corrosion resistance. More than 50% of the Boeing 787 Dreamliner and Airbus 350XWB aircrafts are made of composites [1,2]. However, it is inevitable that an aircraft will encounter lightning strikes during flights. According to statistics published by the Royal Canadian Air Force, a commercial plane is struck by lightning once a year on average, and the probability increases in thunderstorm-prone areas [3,4,5]. When a lightning strike occurs, up to 200 kA of electricity can be generated and released in microseconds [6,7,8]. Due to insulating resins that exist in CFRPs, the conductivity of CFRPs is much lower than that of metallic materials, and it is impossible for CFRPs to dissipate the current from a lightning strike in a short time [9]. The lower electrical conductivity of the CFRP also means that the composites absorb more of the energy from the lightning strike, in the form of resistive heating, which can lead to the catastrophic damage of the composite structures [10,11,12,13].

Constructing a finite element modeling (FEM) model is helpful for guiding the design of CFRP structures to minimize damage to aircrafts from lightning strikes. As such, numerical simulations of the lightning strikes on CFRPs have been widely employed. For example, Ogasawara et al. [14] used the commercial finite element analysis software Abaqus to simulate the lightning-induced electrothermal coupling effects in carbon fiber composites. However, the invariable parameters used in these simulations lead to inaccurate results that suggested a high-temperature region is induced inside the composites rather than at the injected points. Chippendale et al. [15] studied the degradation of composites by electrothermal pyrolysis using laser ablation experiments. Although the experiment confirmed that the material properties are sensitive to lightning damage, the results did not provide a relationship between the actual material parameters and the electrothermal damage. Based on the model proposed by Ogasawara et al., Abdelal et al. [16] introduced new thermophysical performance parameters that changed with temperature during the simulation. However, lightning damage is an instantaneous and unsteady ablation process, and the associated extremely high temperatures cause direct ablation of materials, leading to phase transformations of the materials and associated changes in their intrinsic properties. Therefore, unlike a thermal decomposition process that occurs at a constant rate, temperature-dependent thermophysical properties cannot accurately predict lightning damage to composites. Liu et al. [17] simulated the damage to composites in an electromagnetic environment. Their results showed that the electromagnetic field had a minimal effect on the composites. Fu et al. [18] studied the damage to composites during lightning strikes from the perspective of electrical breakdown. The research indicated that current accumulation from the lightning strike is the main cause of the composite breakdown. Dong et al. [19] proposed an electrical–thermal-pyrolytic model using numerical calculations of the degree of resin pyrolysis that is based on thermal–electrical numerical analysis. This research suggested that the degree of resin pyrolysis is a good indicator of the extent of lightning damage and is a reasonable indicator for both numerical and experimental results.

Based on these simulation results, it can be concluded that modeling the high-resistance heating induced by lightning strikes is relevant to CFRP structures and properties. Generally, the electrical and thermal energy from the lightning strike is conducted along the fibers, while severe material damage occurs in the transverse direction. However, the conductive properties of the material become more complex, when the structure of the CFRP is destroyed by lightning damage. Based on the previous work mentioned above, future studies are needed to clarify the mechanisms of lightning damage in CRFP materials and build the relationship between the lightning damage and the parameters of CFRP structures and properties for designing lightning protection technologies.

In this paper, a revised finite element model was established in the ABAQUS (2017 Edition, Dassault Systemes, France) to analyze the thermal–electrical coupling in composite laminates with the imposed current of a lightning strike. The established simulation model was revised according to the experimental results for lightning strike damage. Finally, the effects of the structure and properties of the composite laminate on the extent of damage were analyzed to predict possible lightning protection solutions.

## 2. Finite Element Model

### 2.1. Theory

Coupled thermal–electrical analyses were conducted using commercial finite element analysis software. The electrical potential on the composite from the lightning strike was simulated for a given electrical boundary condition. Joule heat generation was determined from the electrical to thermal energy conversion based on the simulated transient heat transfer analysis. The thermal and electrical damage from the Joule heat generation process was calculated for each finite element.

The electric field in each grid unit was calculated from the Maxwell equation and shown as:

(1) $${\int^{\;}}_{S}JndS={\int^{\;}}_{V}r_{c}dV,$$

where *V* is the unit volume, *J* is the unit current density, *S* is the unit cross-sectional area, *r_c_* is the unit current volume density, and *n* is the outward normal to *S*. Using this definition of the electrical field, the current density can be described with Ohm’s law:

(2) $$J=\sigma^{E}\times E=-\sigma^{E}\times \frac{\partial_{\varphi }}{\partial_{x}},$$

where *σ^E^* is the electrical conductivity, *E* is the electrical field, and *φ* is the electrical potential.

The Joule heat *P_ec_* can be calculated according to Joule’s law as:

(3) $$P_{ec}=J\times E=\frac{\partial_{\varphi }}{\partial_{x}}\times \sigma^{E}\times \frac{\partial_{\varphi }}{\varphi_{x}}=E\times \sigma^{E}\times E.$$

The average Joule heat ${\bar{P}}_{ec}$ over the entire time of the lightning strike can be expressed as:

(4) $${\bar{P}}_{ec}=E\times \sigma^{E}\times E-E\times \sigma^{E}\times ∆E+\frac{1}{3}∆E\times \sigma^{E}\times ∆E,$$

where *E* and *σ^E^* are the electrical field and electrical conductivity at time t, respectively, and the heat energy converted from the electricity can be written as:

(5) $$Q_{v}=\eta_{v}\times P_{ec},$$

where the *η_v_* is the energy conversion factor.

Therefore, the temperature field for the lightning damage model included lightning Joule heat generation, heat transfer, and dissipation. According to the energy balance, the heat conduction can be summarized as:

(6) $${\int^{\;}}_{V}\rho C_{V}\frac{\partial \theta }{\partial t}\delta \theta dV+{\int^{\;}}_{V}\nabla \delta \theta \times \left(k\times \nabla \theta \right)dV={\int^{\;}}_{V}\delta \theta rdV+{\int^{\;}}_{S}\delta \theta qdS,$$

where *ρ*, *θ*, *C_v_*, *k*, *q,* and *r* represent the density, temperature, specific heat, thermal conductivity, heat flux per unit area, and heat generation density, respectively.

### 2.2. Numerical Model

**Figure 1:**
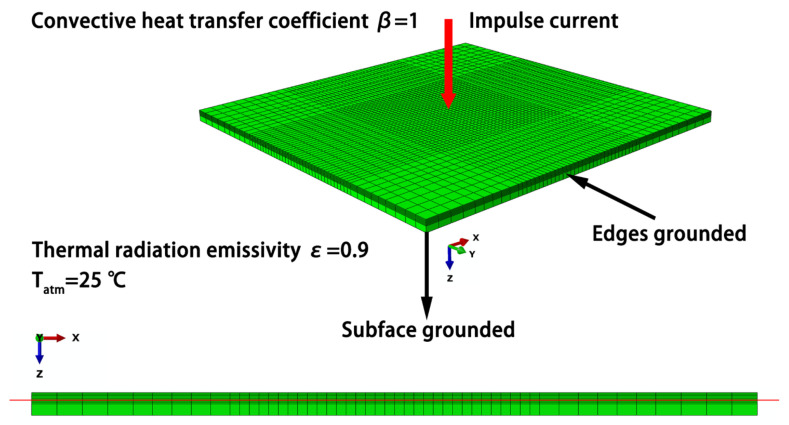
Schematic diagram of the composite lightning damage model. The red line represents the damage depth of 1.6 mm.

The lightning strike damage model for the CFRP was created using the commercial software ABAQUS, as shown in Figure 1. The model was based on a quasi-isotropic laminate with the sequence of [45/0/–45/90]_2s_. The dimensions of the model were 150 × 150 × 4.6 mm^3^, and the thickness of each layer was set to 0.2875 mm. As the cross-section view shown in Figure 1, the mesh size in the thickness direction is the superposition of the thickness of each layer, instead of being divided according to the total thickness. The bottom 8 layers were simplified as an integrated layer to reduce the number of calculations, whereas the top half of the model was designed according to different laminate sequences, which was verified in simulations where the damage depth of composites was approximately 1.6 mm (5–6 layers) at 40 kA. The lightning current was injected into the model at the center node of the top surface using a node-loading form. The model was divided into a DC3D8E grid type, and a total of 23,868 solid elements were used to simulate the electrical–thermal propagation. The lightning current was injected into the model at the center node of the top surface, and the voltage was presumed to be zero at the subface and four side surfaces. 

When the lightning current was injected, the heat was immediately transferred through the composite. Consequently, the thermal emissivity of the entire composite surface was set to 0.9, the convective heat transfer coefficient was 1, and the ambient temperature was 25 °C. The ablation behavior of the resin was considered a decay process, with a degree of pyrolysis *C*:

(7) $$C=\frac{w_{i}-w}{w_{i}-w_{f}}.$$

In Equation (7), *W* is the sample quantity; and *i* and *f* represent the initial and final states of the sample, respectively. Properties of each unit were modeled as a function of *C*, and the epoxy resin was assumed to be almost completely ablated at about 600 °C based on previous thermogravimetric analysis results evaluated from 25 °C to 1000 °C with a constant heating rate of 15 K/min. The thermal degradation was assumed to occur primarily in the carbon fibers. Therefore, the properties of the model CFRP after a degree of pyrolysis of 1 were assigned based on the measured material properties above 600 °C [20,21]. The specific thermo–electro–mechanical parameters are shown in Table 1 and Table 2.

However, the heating rate of the composites is extremely fast during lightning strikes, and resin matrix decomposition cannot be treated as an isothermal process [22], implying that the *C* value calculated from isothermal thermogravimetric analysis before 600 °C is inaccurate. Therefore, this study simulated the thermal degradation of the composite using kinetic equations, and the thermal degradation process was described as a n-level chemical kinetic rate equation as follows [23]:

(8) $$dC/dt=k\left(T\right)\times {\left(1-C\right)}^{n},$$

where *t*, *n*, and *T* are the pyrolysis time, reaction order of the pyrolysis process, thermodynamic temperature, respectively, and *k(T)* is the kinetic rate constant determined from the Arrhenius equation and related with activation energy *E_a_*:

(9) $$k\left(T\right)=Aexp\left(-E_{a}/RT\right),$$

where *A* and *R* are the pre-exponential factor and gas constant, respectively.

**Figure 2:**
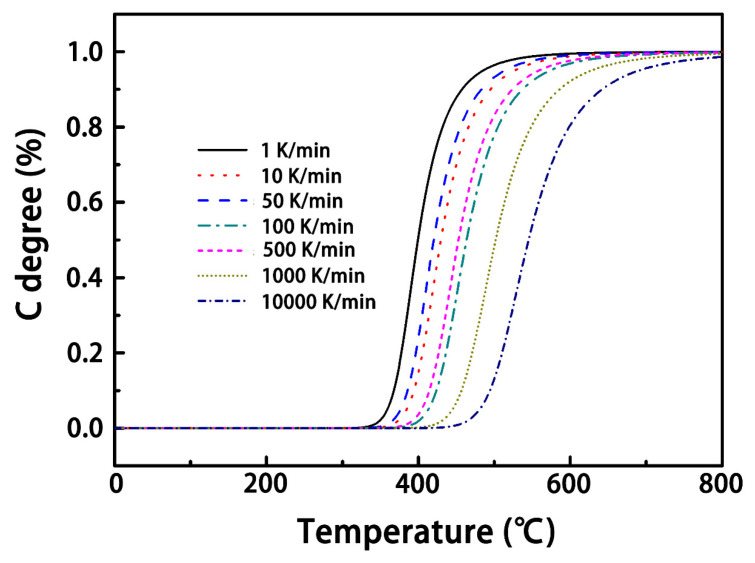
Degree of pyrolysis versus temperature at different heating rates.

According to data published in the work by Ogasawaral et al., [14], the parameters used in the simulations were defined as *n* = 3.5, *A* = 3.0 × 10^15^ s^−1^, *E_a_* = 180 J/(mol·K), and *R* = 8.314 J/(mol·K). Figure 2 displays the simulated results for the resin ablation process at different heating rates using the chemical kinetics equation. It can be found that the shapes of the degree of pyrolysis curves at different heating rates were basically identical, but the onset temperature increased with increasing heating rates. Therefore, the onset temperature can be used to revise the non-isothermal chemical kinetics equation to better match the conditions of lightning strikes, as discussed below.

According to 30 kV/mm of the electrical breakdown strength of epoxy resin [24], the electrical breakdown of the composite with a 55% fiber content was set as 13.5 kV/mm. When the electrical breakdown intensity of grids was beyond 13.5 kV/mm, the electrical properties of the grids were converted to 0.1 S/mm.

**Figure 3:**
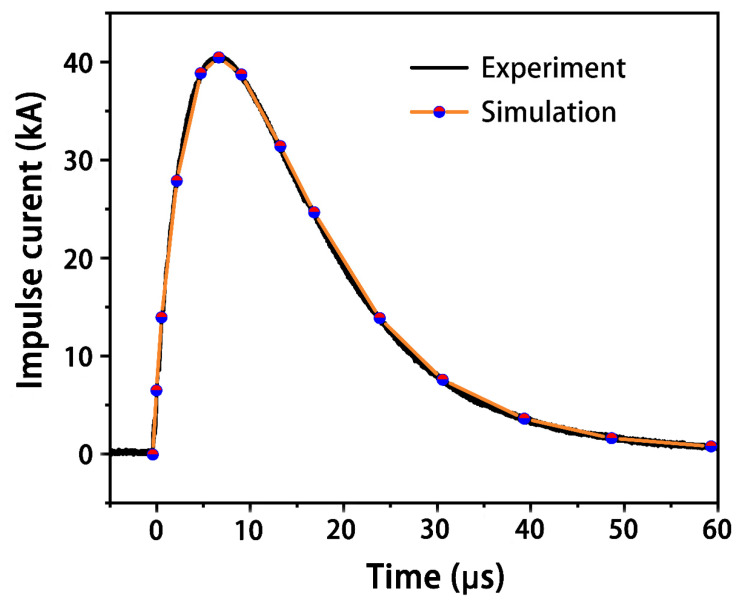
Experimentally applied current of component A.

In addition to resin ablation by the current, the sublimation temperature of the carbon fibers was set to 3316 °C by transforming the latent heat from 4.8 × 10^3^ kJ/kg to 4.3 × 10^4^ kJ/kg [16]. The lightning current waveform of component A in SAE ARP5412 with a modified peak of 40 kA was used in the experimental lightning tests on the CFRP specimens. According to this waveform, the corresponding theoretical waveform used in the present numerical model is plotted in Figure 3. 

**Figure 4:**
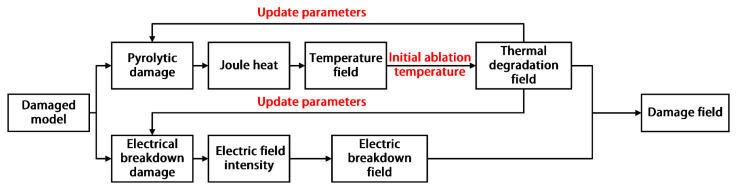
Flow chart of the calculation for the lightning damage model.

As shown in Figure 4, the user-defined subroutines USDFLD and HEAVEL were employed to describe the thermal degradation field and electrical breakdown field, respectively. In each analytical step, the transient electrical and the thermal field analysis were performed sequentially. In the calculative process, the superposition calculations began with the degree of thermal degradation when the thermal field reached the initial ablation temperature value, and then the corresponding thermoelectric properties of the material for the given degree of thermal degradation were updated accordingly in each analysis step. Eventually, the obtained final model results for the degree of lightning damage were the thermal degradation field and the electrical breakdown field.

**Figure 5:**
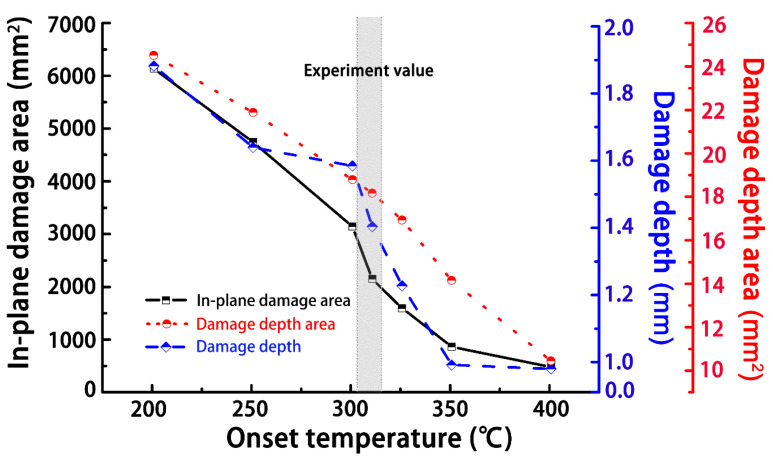
Simulation results from the damaged area and depth with varying values for the onset temperature of resin ablation and comparison with experimental results.

**Figure 6:**
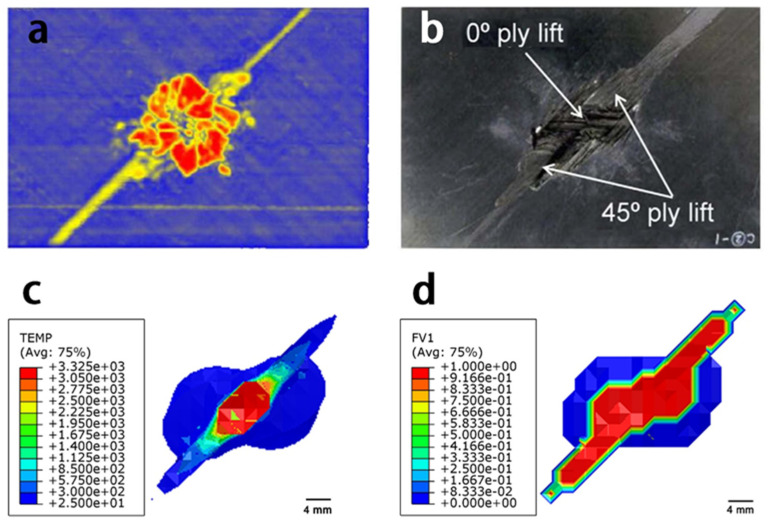
Comparison of the in-plane damage from experimental and simulated results: (**a**) ultrasonic C-scan result; (**b**) surface damage result [25]; (**c**) simulated in-plane damage results from the isothermal pyrolysis diagram; and (**d**) non-isothermal pyrolysis method.

**Figure 7:**
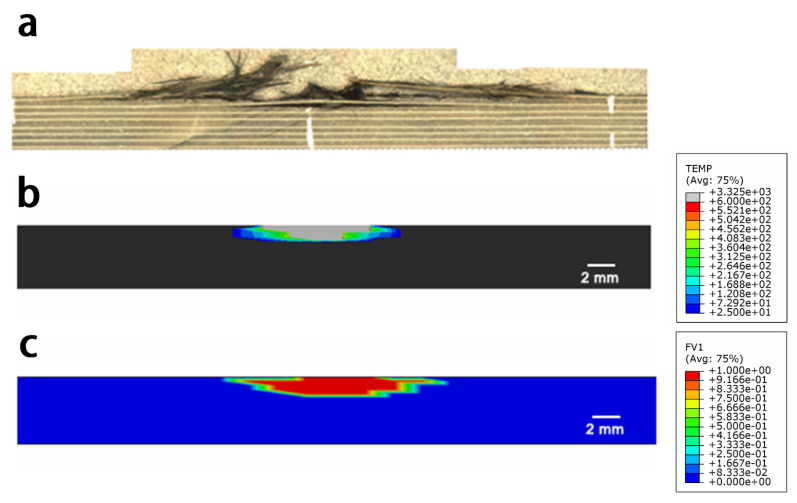
Comparisons between the experimental and simulated results for the depth damage: (**a**) depth damaged area after lightning strike [21]; (**b**) simulated depth damage results from the isothermal pyrolysis diagram; and (**c**) non-isothermal pyrolysis method.

Figure 5 shows the simulated damage to the material with different onset temperatures of resin ablation. The in-plane damage area included the superposition of surface damage in each layer, which corresponded to the ultrasonic C-scan result; the depth damage area represented the damage area along the thickness direction, which corresponded to the ultrasonic B-scan result. The damage depth claimed the maximum damage depth in the thickness direction. In particular, the simulated damage results agreed well with the experimental results, when the simulated onset temperature was 320 °C. A detailed morphology comparison can be found in Figure 6 and Figure 7 that the simulated in-plane and depth damage morphologies were directly compared with experimental results in reference [25]. In addition, the simulation setting condition corresponded to the experimental setup from reference [25]: the subface of the specimen was earth-grounded, and the distance between the tip of the discharge probe and the specimen surface was adjusted to about 2.0–3.0 mm. The lightning current waveform of t1/t2 of 4/20 μs with a peak of 40 kA was conducted in the experiment. The simulated results are in good agreement with the experimentally measured damage morphologies, indicating that Joule heating was the primary source of lightning damage to the composite. The red and blue regions of the diagram represent the fully and slightly decomposed regions of the composite, respectively, with *C* values between 0 and 1. However, other factors such as shockwaves, thermal expansion, pyrolysis gas, and electrical breakdown could result in further damage to the composite. Thus, the experimental damage is expected to be more severe than the simulated results [19]. Nevertheless, the non-isothermal pyrolysis method adopted in this study still showed superior simulated results compared to previous work based on isothermal pyrolysis results. Basically, the simulated result from the non-isothermal pyrolysis method predicted more severe damage. In particular, the simulated severely damaged region is closer to the experimentally observed morphology, because this non-isothermal pyrolysis method involves the overall consideration of the temperature-, time-, and space-dependent material properties as a result of a lightning strike.

## 3. Results and Discussions

### 3.1. Influence of the Composite Structural Parameters on the Extent of Lightning Damage

The injected current from the lightning strike was primarily dissipated along the conductive fiber in the CRFP. In addition, changes in the structural geometry of the composite and laminate material sequences also determined the length of the path to the ground. In this section, the structural geometry parameters that can be tuned to improve the internal current export from the CRFP to reduce the associated lightning damage are discussed.

#### 3.1.1. Thickness

**Figure 8:**
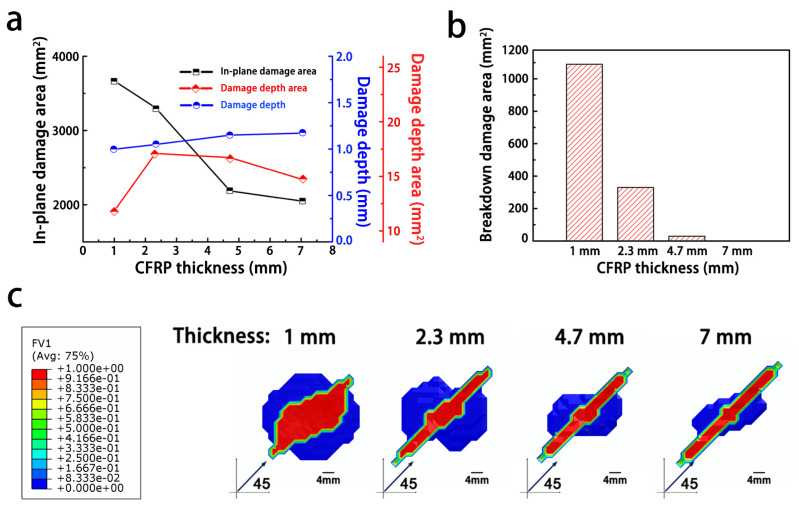
The influence of the composite thickness on the degree of lightning damage to the composites: (**a**) degree of damage to the composites; (**b**) breakdown area of the composites; (**c**) damaged area structure.

Figure 8a,b shows the influence of the thickness of the quasi-isotropic CFRP ([45/0/–45/90]_2s_) on the extent of the lightning strike damage. With an increase in the composite thickness, the size of the in-plane damaged area decreased gradually, while the extent of damage in the thickness direction tended to increase (note: the damage in the sample with a 1 mm thickness penetrated the whole sample). In addition, in the CFRP specimen with a thickness of 7 mm, the breakdown damage area can barely be traced, because the specimen can withstand the voltage surge from the lightning current channel. The damaged area was circular in shape for small thicknesses (Figure 8c). With an increase in the composite thickness, the in-plane damage expanded along the fiber direction, while the damage vertical to fibers direction was gradually reduced.

Because of the insulating resin in the interply, the impedance in the thickness direction of the composite was assumed to decrease as the thickness of the resistive resin decreasing, which resulted in an incremental increase in the injected current in the thickness direction. When the CRFP thickness was small, it can be supposed that more of the injected current was conducted along the thickness direction, and thus, there were a less in-plane current and indistinguishable anisotropic in-plane damage. The simulation results for thinner composites showed evidence of a larger dielectric breakdown area, which supported that the current was concentrated in the attached region of the composite and conducted along the thickness direction. On the contrary, as the thickness increased, the associated greater impedance resulted in more of the current being dispersed along the in-plane fiber direction and more in-plane damage while less damage occurred in the depth direction [25].

#### 3.1.2. Side Length

In contrast to the trends seen with changes in the composite thickness, the in-plane damage decreased, as the side length increased (note: The composite with the side length of 37.5 mm exhibited the fully penetrated in-plane damage).

**Figure 9:**
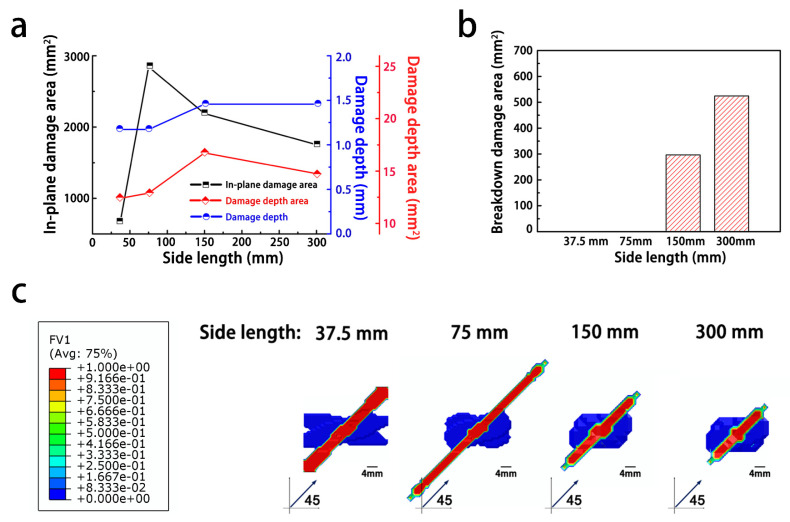
Influence of the side length on the degree of lightning damage to the composites: (**a**) damage degree of composites; (**b**) breakdown area of the composites; (**c**) damage morphology.

Figure 9a,c shows that the degree of damage along the fiber direction first increased and then decreased as the side length increased. This non-monotonic trend was generated, because the current injected on the surface of the composite was not dissipated as the conduction path increased with the increasing side length. As a result, the current accumulated in the area struck by lightning, inducing a strong electrical field and eventually causing a large dielectric breakdown, as shown in Figure 9b.

From the analysis above, it can be found that the structural geometry of the composite mainly affected the current export path. Therefore, it is essential to decrease the length of the export path to minimize the degree of damage to the composite. However, reducing the composite thickness did not fully alleviate the damage, because the low conductivity along the thickness direction led to more severe damage from the current. Due to the electrical conductivity of the carbon fibers, the conduction of the current along the fiber direction was promoted by decreasing the side length of the composite, thus promoting the dispersion of the current from a lightning strike. Therefore, shortening the dispersion path through a strategic structural design is beneficial for reducing lightning damage to composites, which can be applied to the fuel tanks fabricated by the CFRP. In general, subface ground is unfeasible and dangerous for fuel tanks, because it may introduce fuel ignition by lightning current injection. As a result, the distance of the grounded rivets installed in CFRPs should be decreased to shorten the dispersion path of the current. Moreover, thinner composites are more prone to dielectric breakdown.

#### 3.1.3. Laminate Sequence

The electrical properties of different ply sequences can be derived using a transformation matrix R [26]:

(10) $$\left[\;\sigma \;\prime \;\right]=\left[\begin{array}{ccc} \sigma_{11} & 0 & 0\\ 0 & \sigma_{22} & 0\\ 0 & 0 & \sigma_{33} \end{array}\right]$$

(11) $$R=\left[\begin{array}{ccc} cos\alpha & sin\alpha & 0\\ -sin\alpha & cos\alpha & 0\\ 0 & 0 & 1 \end{array}\right]$$

(12) $$\left[\sigma \right]={\left[R\right]}^{-1}\times \left[\sigma^{\prime }\right]\times \left[R\right]$$

(13) $$\left[\sigma \right]=\left[\begin{array}{ccc} \sigma_{11}m^{2}+\sigma_{22}n^{2} & \left(\sigma_{11}-\sigma_{22}\right)mn & 0\\ \left(\sigma_{11}-\sigma_{22}\right)mn & \sigma_{11}n^{2}+\sigma_{22}m^{2} & 0\\ 0 & 0 & \sigma_{33} \end{array}\right]$$

where σ^’^ is the conductivity of the origin ply, and σ is the conductivity of the transformed ply; the subscripts 11, 22, and 33 represent the fiber direction, the in-plane vertical fiber direction, and the thickness direction, respectively; m and n are parameters of the rotation matrix, namely m = cosα and n = sinα (α is the layup angle). This equation suggests that the laminate sequence can be designed to ensure multi-directional conductivity and create more current export paths to improve the internal current dispersion in composites.

To evaluate the influence of the laminate layer sequence on the current dissipation, the angles of the fibers in each layer were varied, namely [0]_16_, [0/30/60/90]_2s_, [0/45/90/–45]_2s_, and [0/90]_4s_. To simplify the labelling scheme, these models are noted using the 2nd layer sequence as 0, 30, 45, and 90, respectively. 

**Figure 10:**
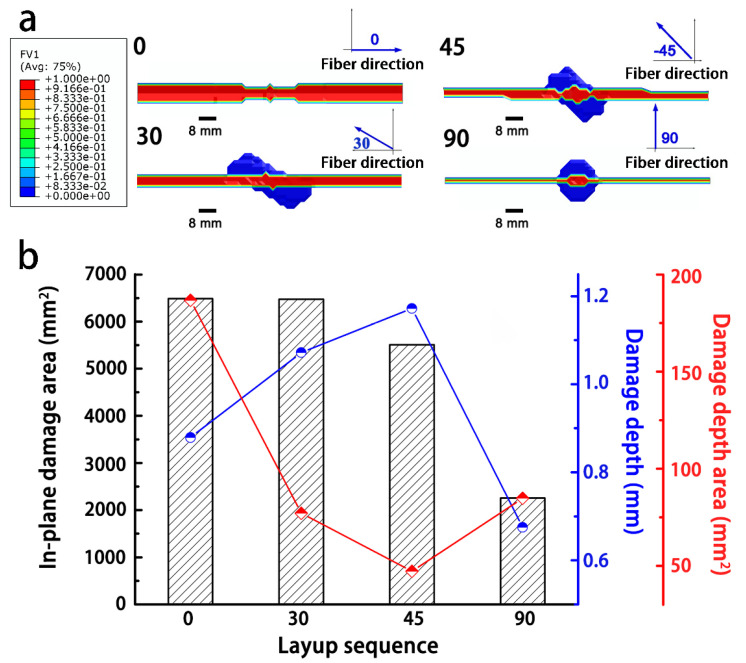
Influence of the layer sequence on the degree of damage from lightning to the composites: (**a**) damaged morphology; and (**b**) degree of damage to the composites.

**Figure 11:**
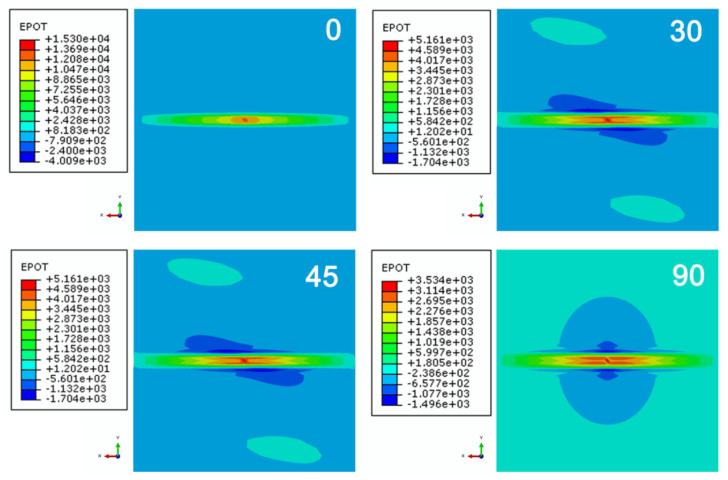
Potential distributions at the surface of the composites with different layup sequences.

The simulation results are shown in Figure 10, and models 0 and 30 had larger damaged areas than the other models. Because the current flow paths in the 1st layers for all models were identical, whereas the current paths in the adjacent 2nd layer distributed along another direction, which was dedicated to varying the current dispersion between adjacent layers. When the angle between adjacent layers increased, the current could disperse more widely, resulting in decreasing the extent of Joule heating. Therefore, the size of the damaged area reduced with an increase in the angle difference between the 1st and 2nd layers, but the depth of the damage in the transverse direction increased at the 1st layer and then dropped. From the perspective of the damage morphology, all the composite models with different layer sequences showed a distribution of morphologies parallel to the fiber direction in the 1st layer. Model 0 exhibited the maximal degree of damage, whereas model 90 showed the least damage. From the discussion above, the distance of the export path was a key factor in determining the Joule heating inside the model. Obviously, models 0 and 90 had the shortest exports paths. However, in model 0, the fibers are aligned in the same direction, resulting in the electrical current concentrating in the fiber direction [27]. From Figure 11, the nephogram of the electrical potential showed that the model 0 composite sequence had a higher concentration of the current compared to those of the other models, thus causing the accumulation of Joule heat in the 0° direction and thereby leading to more severe damage. In contrast, the electrical potential area expanded with the angle difference between the 1st and 2nd layers, resulting in a wider distribution and a lower degree of electrical potential. Therefore, in addition to shortening the current export path, it is essential to disperse the electrical current inside the composite as much as possible to minimize the damage from a lightning strike. A sequence of laminates with orthogonal fibers is recommended to minimize damage from lightning compared to other ply sequences.

### 3.2. Influence of the Composite Properties on the Extent of Lightning Damage

Although many factors can influence the extent of the lightning damage to a composite, the thermal and electrical properties of materials undoubtedly play a dominant role. In this section, anisotropies in the thermal and electrical properties of the composites are discussed.

#### 3.2.1. Electrical Conductivity

**Figure 12:**
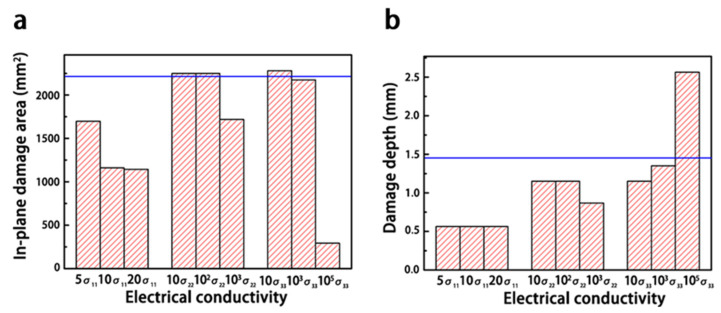
Influence of the electrical conductivity on the degree of lightning damage: (**a**) in-plane damages with different electrical conductivities; (**b**) in-depth damages with different electrical conductivities. The number in front of σ represents the increase in the conductivity of the composite, and the blue line represents the degree of damage in the original composite.

**Figure 13:**
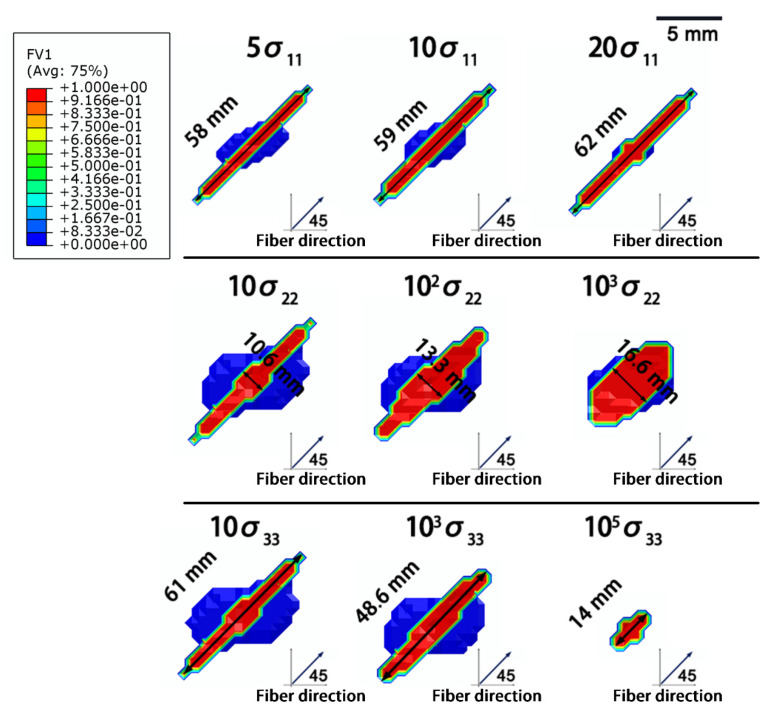
Morphologies of the in-plane damage in composites with different electrical conductivities.

As shown in Figure 12, increasing the electrical conductivity along the fiber direction (σ_11_) effectively enhanced the current dissipation in the composite and reduced the extent of the in-plane damage, but the damage along the depth of the composite was unchanged. The damage was concentrated along the fiber direction, and the length of damage extended with increasing σ_11_ (Figure 13). Because the increased electrical conductivity facilitated the current propagation along the fiber direction, a lower current was conducted along other directions in the composite [18,28,29].

Enhancing the electrical conductivity along the vertical fiber direction (σ_22_ and σ_33_) had little effect on the simulated lightning damage, until σ_22_ and σ_33_ were close to the value of σ_11_. For instance, the extent of damage did not sharply improve, until σ_22_ increased up to 1000 times (1.145 × 10^3^·S/m). Because a slight improvement in the electrical conductivity along the transverse fiber direction did not affect the current conduction in the laminate, the current was still mainly dissipated along the fiber direction. When the electrical conductivity along the transverse direction was close to σ_11_, the current was transported along more paths. Consequently, the damage was more homogeneous, and the overall degree of damage was lower.

#### 3.2.2. Thermal Conductivity

**Figure 14:**
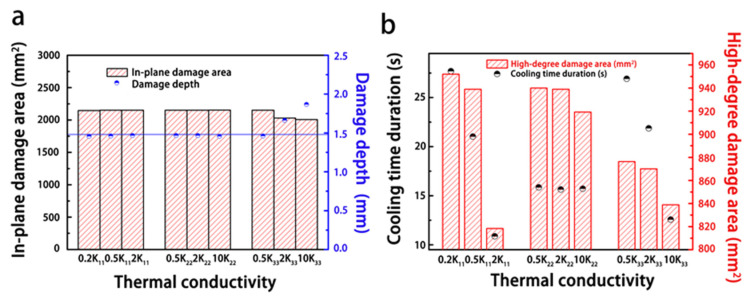
Influence of the thermal conductivity on the extent of the lightning damage: (**a**) variation in the degree of lightning damage with varying thermal conductivities (**b**) the relationship between the thermal conductivity, cooling duration, and size of the highly damaged area. The blue line represents the degree of damage in the original composite, and the number in front of thermal conductivity (K) indicates the increment multiple.

Figure 14 reveals the influence of the thermal conductivity of the composite on the resulting lightning damage. It can be found that the thermal conductivity had a minimal effect on the degree of lightning damage. Because the lightning process is instantaneous, even if thermal conductivity increased by orders of magnitude, the high current conduction still resulted in significant amounts of Joule heating in microseconds, and heat dissipation in such a short time hardly affected the extent of the damage. However, increasing the thermal conductivity of the composite did promote thermal dissipation after the lightning strike, and as a result, the heat was less concentrated and cooling time was faster in the highly damaged areas near the lightning strike point (Figure 14b).

**Figure 15:**
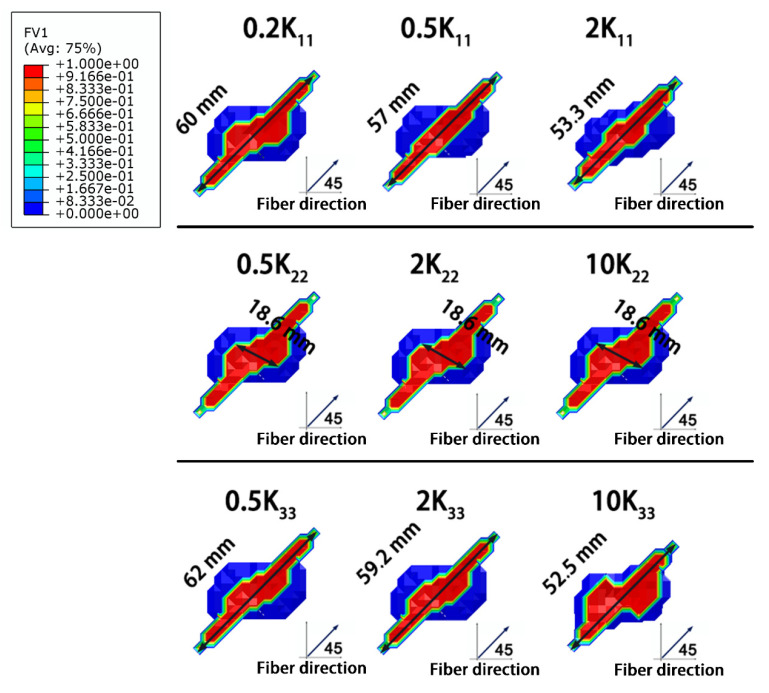
Morphologies of the lightning damage to composites with varying thermal conductivities.

In Figure 15, because the high-temperature region induced by the Joule heating was distributed along the fiber direction, increasing the thermal conductivity along the fiber direction (K_11_) facilitated thermal conduction. Thus, high temperature was dissipated in shorter cooling durations, and thereby the size of the highly damaged area decreased with increasing K_11_. In contrast, increasing the thermal conductivity vertical to the fiber direction (K_22_) did not affect the heat transfer path. As a result, the size of the damaged area did not change with changes in K_22_. Increasing the conductivity along the thickness direction (K_33_) reduced the degree of the lightning damage. Heat transfer along the thickness direction is the shortest path compared to the other directions. Therefore, improving K_33_ promoted rapidly heat dissipation along the thickness direction, as seen in Figure 14a, where the depth of damage increased with increasing K_33_ as more heat was conducted along the thickness direction.

#### 3.2.3. Specific Heat Capacity

**Figure 16:**
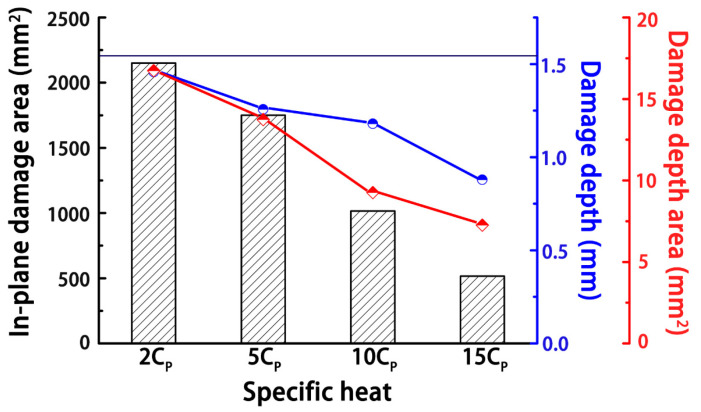
Influence of the specific heat capacity on the degree of lightning damage to the composites. The number in front of the specific heat capacity (C_p_) indicates the number of the increment, and the black line represents the degree of damage in the original composite.

As shown in Figure 16, the specific heat capacity of the composite determined the amount of heat energy required to raise the temperature of the material per unit of mass, and the composite was supposed to be more thermally stable as the specific heat capacity increased. Thus, the degree of damage was effectively reduced, as the specific heat capacity of the composite increased. However, to be effective against a lightning strike, the heat capacity needed to be as high as 10 kJ/(kg·K), and only a few gases had such high specific heat capacities. Therefore, increasing the specific heat capacity of solid materials is very hard to realize.

## 4. Conclusions

The effects of structural geometry, laminate sequences, and intrinsic properties of composites on the degree of lightning damage were studied using numerical simulations, and the main conclusions were as follows:

1. The structural geometry of the composite determines the dissipation path of the current from a lightning strike. To improve the lightning strike protection, it is necessary to reduce the distance of the export path. In addition, the sequence of the laminates in the composites can affect the current dispersion and the export distance.

2. The current export path should be minimized in the design of composite structures for lightning protection. The sequence of [0/90]_4s_ is optimal for dispersing electrical potential and shortening the current export path.

3. The thermal and electrical conductivities are strongly influenced by the anisotropic structure of the composite. In terms of the fiber direction, improving the conductivity can facilitate thermal or electrical conduction to reduce the Joule heating. The conductivity along the transverse fiber direction should be increased to realize lightning current or energy path transformation.

4. Improving the electrical conductivity is a more effective means of enhancing the lightning protection of a composite than improving the thermal conductivity. Improving the electrical conductivity requires enhancing the current export path, whereas improving the thermal conductivity is beneficial for dissipating the heat after lightning strikes.

This work aimed to provide a reference for the design of composites for better lightning strike protection. In future work, experiments should be performed to determine if the predicted structural designs and property improvements better protect composite materials against lightning damage. Moreover, the presented work serves as a reference for the optimization of approaches to protect aircrafts from lightning damage.

## Figures and Tables

**Table 1:** Thermal parameters of the composites [20,21].

Temperature (°C)	*Cp* (J/kg·K)	$\alpha_{xx}^{T}$ (1/k)	$\alpha_{yy}^{T}$ (1/k)	$\alpha_{zz}^{T}$ (1/k)	$k_{xx}^{T}$ (W/m·K)	$k_{yy}^{T}$ (W/m·K)	$k_{zz}^{T}$ (W/m·K)	C
25	1065	1.8 × 10^-8^	2.16 × 10^–5^	2.16 × 10^–5^	11.8	0.609	0.609	0
600	4200	5.4 × 10^–8^	3.78 × 10^–5^	3.78 × 10^–5^	1.736	0.1	0.1	1
1168	1800	5.4 × 10^–8^	3.78 × 10^–5^	3.78 × 10^–5^	1.736	0.1	0.1	1
3316	2510	5.4 × 10^–8^	3.78 × 10^–5^	3.78 × 10^–5^	1.376	0.1	0.1	1
>3316	5875	5.4 × 10^–8^	3.78 × 10^–5^	3.78 × 10^–5^	0.105	0.105	1 × 10^3^	1

Note: *Cp* is the specific heat capacity; $\alpha^{T}$ is the coefficient of thermal expansion; *k* is the thermal conductivity; xx, yy, and zz represent the in-fiber direction, vertical fiber direction, and thickness direction, respectively.

**Table 2:** Electrical parameters of the composites [20,21].

Temperature (°C)	$\sigma_{xx}$ (S/m)	$\sigma_{yy}$ (S/m)	$\sigma_{zz}$ (S/m)	C
25	3.6 × 10^4^	1.145	3.9 × 10^−3^	0
600	3.6 × 10^4^	1.145	3.9 × 10^−3^	1
1168	3.6 × 10^4^	2 × 10^3^	2 × 10^3^	1
3316	3.6× 10^4^	2 × 10^3^	2 × 10^3^	1
>3316	1 × 10^6^	2 × 10^3^	2 × 10^3^	1

Note: σ is the conductivity of the composite; xx, yy, and zz represent the fiber direction, vertical fiber direction, and thickness direction, respectively.

## Data Availability

The data presented in this study are available upon reasonable request from the corresponding author.
